# Cardiovascular events and death after myocardial infarction or ischemic stroke in an older Medicare population

**DOI:** 10.1002/clc.23160

**Published:** 2019-02-25

**Authors:** Suying Li, Yi Peng, Xinyue Wang, Yi Qian, Pin Xiang, Sally W. Wade, Haifeng Guo, J. Antonio G. Lopez, Charles A. Herzog, Yehuda Handelsman

**Affiliations:** ^1^ Hennepin Healthcare Research Institute Chronic Disease Research Group Minneapolis Minnesota; ^2^ Amgen Inc. Thousand Oaks California; ^3^ Wade Outcomes Research and Consulting Salt Lake City Utah; ^4^ Hennepin County Medical Center Cardiology Minneapolis Minnesota; ^5^ University of Minnesota Department of Medicine Minneapolis Minnesota; ^6^ Metabolic Institute of America Endocrinology Tarzana California

**Keywords:** aging and the cardiovascular system, atherosclerotic cardiovascular disease, diabetes, Medicare, recurrent event

## Abstract

**Background:**

Survivors of myocardial infarction (MI) or ischemic stroke (IS) are at high risk for subsequent cardiovascular events.

**Hypothesis:**

Older patients with prior MI or IS are at risk for recurrent cardiovascular events, and comorbidities such as diabetes may increase this risk.

**Methods:**

Two cohorts were studied in a retrospective Medicare 20% random sample—a 2008 cohort with up to 6 years of follow‐up (MI, N = 26 460; IS, N = 17 566) and a 2012 cohort with 1 year of follow‐up (MI, N = 26 548; IS, N = 17 728).

**Results:**

In older patients who survived an event of MI or IS (2012 cohort), 7.2% had a recurrent MI and 6.7% had a recurrent IS in the first year; 32% died. Accounting for multiple recurrent events (2012 cohort), the event rates per 100 patient‐years were 11.6 and 10.2 for the MI and IS cohorts, respectively. Cumulative incidence of recurrence (2008 cohort) increased from 7.7% at 1 year to 14.3% at 6 years for recurrent MI and from 6.7% at 1 year to 13.4% at 6 years for recurrent IS. Comorbid diabetes (2012 cohort) was significantly associated (adjusted risk ratio) with MI recurrence (1.44) and risk of coronary revascularization (1.23) in the MI cohort and with IS recurrence (1.26) in the IS cohort.

**Conclusion:**

In this older population with prior MI or IS, high rates of recurrent cardiovascular events and multiple recurrent events were observed. These findings highlight the need for aggressive intervention for secondary prevention and management of comorbidities in high‐risk patients, particularly those with diabetes.

## 1 INTRODUCTION

The burden of cardiovascular disease (CVD) in the United States is high, with 92.1 million adults (46.7 million aged at least 60 years) experiencing at least one cardiovascular event during 2011 to 2014; 7.9 million experienced myocardial infarction (MI) and 7.2 million experienced stroke.^1^ In 2014, CVD accounted for 30.8% of all deaths.^1^ In addition, CVD is the most costly chronic disease in the United States, with estimated direct costs projected to increase from 318 billion dollars in 2015 to 749 billion dollars by 2035.^2^ The preponderance of this cost burden is projected to be attributable to patients at least 65 years of age (450 and 200 billion dollars for individuals ≥80 and 65 to 79 years old, respectively, by 2035).^2^

Survivors of MI or stroke are at high risk for recurrence, other cardiovascular events, or death. For example, a recent analysis of the FOURIER trial demonstrated that high‐risk patients with a history of CVD and maximally tolerated statin therapy are at substantial risk for the recurrence of multiple cardiovascular events.^3^ Among survivors of first MI between 2004 and 2010 in England, recurrence rates were 5.6% for men and 7.2% for women at 1 year, and 13.9% and 16.2%, respectively, at 7 years.^4^ Every year, approximately 795 000 people in the United States experience a stroke, 23.3% of which are recurrent events.^1^ Other studies of stroke recurrence have reported cumulative recurrence rates of 8.0% to 12.6% at 1 year, 10.8% to 12.1% at 2 years, and 16.6% at 5 years.^5^, ^6^, ^7^, ^8^ Among patients surviving a stroke, the incidence of all‐cause death was 24.5% at 1 year and 41.3% at 4 years.^5^ Moreover, healthcare costs in Medicare beneficiaries who have a recurrent CVD event following an MI are 2‐fold greater compared with the period prior to the initial event.^9^

Older age and presence of diabetes are known risk factors for CVD.^1^, ^4^, ^10^, ^11^, ^12^ CVD prevalence rates of 70% and 86% for patients aged 60 to 79 years and 80 years or older have been reported. Mortality rates, both all‐cause and CVD‐related, for patients with diabetes are approximately 2‐fold higher than those for patients without diabetes, and increasing duration of diabetes is associated with increasing CVD risk.^13^, ^14^ Although it is important to understand the burden of CVD in these high‐risk populations, few studies have systematically examined the real‐world pattern of recurrent cardiovascular events in older patients overall or by diabetes status. A recent systematic literature review found that the rates of secondary major adverse cardiovascular events among patients with atherosclerotic CVD were greater in real‐world studies compared with randomized clinical trials; however, this study did not investigate the risk in elderly patients or patients with diabetes.^15^ We estimated cumulative recurrence rates of MI and ischemic stroke (IS) after hospital discharge and cumulative incidence of other cardiovascular events, all‐cause death, and composite measures of multiple events after MI or IS over long‐term follow‐up in a 2008 cohort of older patients. We also examined the association between diabetes status and 1‐year risk of MI or IS recurrence and the incidence of other CVD outcomes in a 2012 cohort.

## 2 METHODS

We analyzed a 20% random sample of Centers for Medicare & Medicaid Services (CMS) Medicare data from 2007 to 2013, which provides a representative sample of all Medicare beneficiaries with fee‐for‐service coverage of Medicare Parts A, B, and D. CMS selects the random sample based on the last four digits of beneficiaries' social security numbers. The study population included Medicare beneficiaries with an index event in 2008 or 2012. The 2008 cohort allowed for quantification of the incidence over long‐term follow‐up (up to 6 years), and the 2012 cohort provided results reflecting more recent clinical practice. We examined two disease cohorts within the 2008 and 2012 study cohorts—patients who experienced MI and patients who experienced IS; the cohorts were not mutually exclusive. The International Classification of Diseases, Ninth Revision, Clinical Modification (ICD‐9‐CM) diagnosis codes and disease definition algorithms used to identify patients with MI, IS, and diabetes are detailed in Appendix SA, Supporting Information. For example, a patient with MI was identified by at least 1 inpatient claim with ICD‐9‐CM code 410 (but not 410.x2) in a cohort year.

Patients were required to meet the following inclusion/exclusion criteria: (a) at least 66 years old as of index date, (b) Medicare Part A (hospital insurance), B (medical insurance), and D (prescription drugs) coverage on index date and for at least 1 year prior to index date, (c) alive on index date, and (d) fee‐for‐service insurance coverage and no end‐stage renal disease prior to index date.

Medicare data include enrollment information, demographic characteristics, and medical claims from Parts A, B, and D. The Medicare claims files contain information collected by Medicare to allow payment for healthcare services provided to Medicare beneficiaries in the United States and its territories. Standard analysis files (SAFs) generated by the CMS were used. SAFs are available for each Part A institutional claim type (inpatient, outpatient, skilled nursing facility, home health agency, carrier, or hospice). Non‐institutional Part B physician/supplier SAFs include final action claims for physician services, laboratory services (not laboratory values), and durable medical equipment. CMS Part D data include the prescription drug event file, which contains the National Drug Index code for each drug, prescription dosing information, and drug costs.

Using a retrospective observational cohort study design, two study cohorts (MI and IS cohorts) were defined for 2008 and 2012 (Figure S1 and Appendix SA). The MI index date was defined as the earliest discharge date in the year of the index event. A baseline period of 1 year before the index date, including the index date, was used to define comorbid conditions. The follow‐up period was from the date after the index date until the end of Medicare Part A, B, or D coverage, death, or December 31, 2013. Cardiovascular events and all‐cause death were defined during the follow‐up period. The same approach was applied to define the 2008 and 2012 IS cohorts.

Outcome definitions during follow‐up are detailed in Appendix SB. Single‐event outcomes included MI, IS, unstable angina (UA), transient ischemic attack (TIA), coronary artery bypass grafting (CABG)/percutaneous coronary intervention (PCI), and all‐cause death. Composite outcomes included (a) MI, IS, or UA; (b) MI, IS, UA, or death; (c) MI, IS, UA, or CABG/PCI; and (d) MI, IS, UA, CABG/PCI, or death.

For each MI and IS study cohort in 2008 or 2012, we calculated the percentages of patients with various outcomes and multiple event rates during the 1‐year follow‐up. Event rates that included multiple events are expressed as number of events per 100 patient‐years. For each disease cohort in 2008, we estimated the cumulative incidence of outcome variables for the 6‐year follow‐up period using the Kaplan‐Meier method. For each non‐death outcome, we estimated the cumulative incidence function to treat death as a competing event.

To examine associations between diabetes status and rates of cardiovascular events and all‐cause mortality in the 2012 MI and IS cohorts, we used the Fine and Gray model incorporating death as a competing risk event to estimate the relative risk (diabetes vs non‐diabetes) for outcomes not including death as a component.^16^ For outcomes including death as a component, we used Cox regression to estimate the relative risk (diabetes vs non‐diabetes). All models were adjusted for age, sex, race, and baseline conditions, including prior CVD events (MI, IS, UA, TIA, or CABG/PCI) and comorbid conditions. Specific comorbid conditions are defined in Appendix SC. Selection criteria and sample sizes for the 2012 MI and IS cohorts are presented in Figure S2.

Qualified researchers may request data from Amgen clinical studies. Complete details are available at the following: http://www.amgen.com/datasharing.

## 3 RESULTS

Eligible patients in the 2012 Medicare 20% random sample included 26 548 patients discharged from hospitalization for MI and 17 728 discharged from hospitalization for IS. For the MI cohort, mean age was 80.1 years, 85.0% were white, and 58.0% were women (Table 1). For the IS cohort, mean age was 80.9 years, 81.7% were white, and 64.4% were women. Hypertension (92.4%), coronary atherosclerosis/angina/old MI (80.5%), and heart failure (56.2%) were the most common baseline comorbidities in the 2012 MI cohort (Table 1). In the IS cohort, the most common baseline comorbidities were hypertension (94.5%), cerebrovascular disease (64.3%), and coronary atherosclerosis/angina/old MI (45.3%).

**Table 1:** Baseline demographics and comorbid conditions: 2012 MI and IS cohorts

Study cohorts	MI (N = 26 548)	IS (N = 17 728)
Demographics		
Age, years, mean (SD)	80.1 (8.3)	80.9 (8.2)
Age group on index date, %		
66‐74 years	32.4	28.0
75‐84 years	37.1	38.1
85 years or older	30.5	33.9
Race, %		
White	85.0	81.7
Black	8.6	11.9
Other/unknown	6.4	6.5
Gender, %		
Male	42.0	35.6
Female	58.0	64.4
Census region, %		
Northeast	20.6	17.3
Midwest	25.4	24.5
South	39.7	43.3
West	14.1	14.7
Missing	0.2	0.2
Comorbidity prevalence, %		
MI (STEMI or NSTEMI)	33.5	16.0
Unstable angina	6.2	2.2
IS	6.8	25.1
Hemorrhagic stroke	1.2	4.8
Cerebrovascular disease	22.3	64.3
TIA	3.2	9.6
Carotid endarterectomy	0.7	2.0
Carotid/vertebral/basilar stenting	0.2	0.6
CABG surgery, PCI	34.6	2.9
PAD	28.7	24.5
PAD with amputation *or* peripheral artery bypass surgery *or* peripheral intervention	2.4	1.4
Aneurysm	4.8	3.3
Endovascular stent/graft	1.9	0.4
Hypertension	92.4	94.5
Dyslipidemia/hyperlipidemia	25.1	24.5
Heart failure	56.2	32.5
Venous thromboembolism	7.1	6.4
Coronary atherosclerosis/angina/old MI	80.5	45.3
HeFH	0.5	0.3
Cancer (excluding non‐melanoma skin cancer)	14.0	12.5
HIV	0.1	0.1
Rheumatoid arthritis	3.9	3.6
CKD stages 1‐5 (not on dialysis or transplant)	49.2	34.8
Carotid/vertebral/basilar stenosis	8.7	22.4
Smoking	33.1	27.0
Obesity^a^	9.5	7.5

Abbreviations: CABG, coronary artery bypass grafting; CKD, chronic kidney disease; HeFH, heterozygous familial hypercholesterolemia; HIV, human immunodeficiency virus; IS, ischemic stroke; MI, myocardial infarction; NSTEMI, non–ST‐elevation MI; PAD, peripheral artery disease; PCI, percutaneous coronary intervention; STEMI, ST‐elevation MI; TIA, transient ischemic attack.

a Obesity was defined by International Classification of Diseases, Ninth Revision, Clinical Modification diagnosis codes: 278.01, 278.03, V85.3x, and V85.4x.

The 2008 study cohorts included 26 460 and 17 566 patients with MI and IS, respectively. Mean (median) follow‐up times were 2.65 (2.15) years for the MI cohort and 2.62 (2.17) years for the IS cohort. Baseline characteristics of the 2008 cohort were similar to those of the 2012 cohort.

In the 2012 MI cohort, 7.2% of the patients experienced a recurrent MI in the first year after the index MI, and the recurrence rate when accounting for multiple MI recurrences was 11.6 per 100 patient‐years (Table 2). In 2012 IS cohort, the incidence of recurrent IS was 6.7% during the first year, and the event rate was 10.2 per 100 patient‐years when multiple IS recurrences were included. Event rates for the composite outcome that included MI, IS, UA, or CABG/PCI were 15.4% (26.7 events per 100 patient‐years) for the 2012 MI cohort and 9.0% (14.4 events per 100 patient‐years) for 2012 IS cohort. Approximately 32% of the patients in each cohort died within 1 year of the index MI or IS.

**Table 2:** Cardiovascular events and all‐cause death during the first year after MI or IS: 2008 and 2012 cohorts

Study cohorts	MI^a^	IS^b^	UA	TIA	CABG/PCI	Death	Composite events			
							MI, IS, or UA	MI, IS, UA, or death	MI, IS, UA, or CABG/PCI	MI, IS, UA, CABG/PCI, or death
Incidence of event (%)										
2008										
MI	7.6	1.7	0.2	0.7	10.0	32.0	9.4	37.7	16.7	44.2
IS	1.9	6.6	0.1	1.9	1.4	32.4	8.4	37.7	9.3	38.5
2012										
MI	7.2	1.7	0.2	0.6	9.3	31.6	8.9	37.2	15.4	42.8
IS	1.6	6.7	–c	1.7	1.4	31.6	8.2	36.8	9.0	37.5
Event rate per 100 patient‐years^d^										
2008										
MI	12.5	2.5	0.3	0.9	14.5	43.2	15.3	58.5	29.8	72.9
IS	2.8	10.1	0.2	2.7	2.2	44.1	13.0	57.1	15.2	59.3
2012										
MI	11.6	2.5	0.3	0.8	12.3	42.2	14.4	56.6	26.7	68.9
IS	2.4	10.2	0.04	2.5	1.8	42.6	12.6	55.2	14.4	57.0

Abbreviations: CABG, coronary artery bypass grafting; IS, ischemic stroke; MI, myocardial infarction; PCI, percutaneous coronary intervention; TIA, transient ischemic attack; UA, unstable angina.

a Recurrent MI for the MI cohort.

b Recurrent IS for the IS cohort.

c Value is suppressed due to smaller event size (10 events or less) according to Centers for Medicare & Medicaid Services reporting rules.

d Multiple event rates for non‐death events.

**Figure 1:**
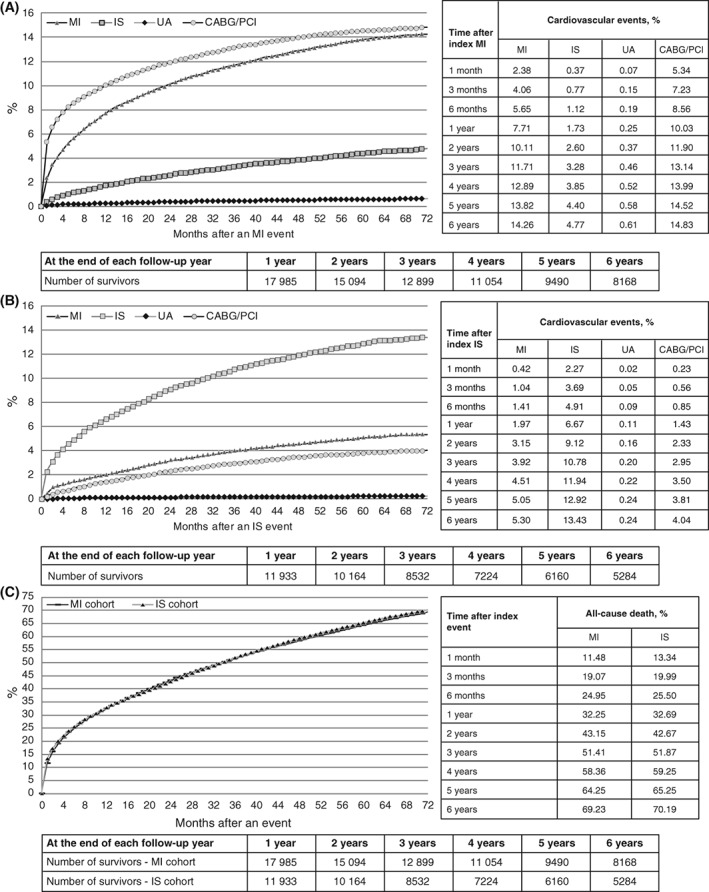
Cumulative incidence of cardiovascular events or death after index MI or IS: 2008 cohort. (A) MI cohort, (B) IS cohort, and (C) all‐cause death. CABG, coronary artery bypass grafting; IS, ischemic stroke; MI, myocardial infarction; PCI, percutaneous coronary intervention; UA, unstable angina

The cumulative incidence of cardiovascular events following an index event for the 2008 cohort is presented in Figure 1 (A, MI cohort; B, IS cohort). After an index MI event, the incidence of MI recurrence was 5.7% at 6 months, 7.7% at 1 year, 11.7% at 3 years, and 14.3% at 6 years. After an index IS event, the incidence of IS recurrence was 4.9% at 6 months, 6.7% at 1 year, 10.8% at 3 years, and 13.4% at 6 years. Among patients in the MI and IS cohorts with 1 year of follow‐up, 9.5% and 8.5%, respectively, experienced the composite outcome of MI, IS, or UA (Table 3). When the composite outcome also included CABG/PCI, rates were 16.9% for the MI cohort and 9.4% for the IS cohort. The cumulative incidence for the composite outcomes increased over time; after 6 years of follow‐up, the cumulative incidence for the MI, IS, or UA composite outcome was 18.9% in the MI cohort and 18.0% in the IS cohort (27.5% and 19.9%, respectively, when CABG/PCI was included).

**Table 3:** Cumulative incidence (%) of composite events after an index event of MI or IS: 2008 MI and IS cohorts

Follow‐up time after index event	MI cohort				IS cohort			
	MI, IS, or UA	MI, IS, UA, or death	MI, IS, UA, or CABG/PCI	MI, IS, UA, CABG/PCI, or death	MI, IS, or UA	MI, IS, UA, or death	MI, IS, UA, or CABG/PCI	MI, IS, UA, CABG/PCI, or death
1 month	2.8	14.0	7.3	18.0	2.7	15.8	2.9	15.9
3 months	4.9	22.7	10.8	28.1	4.6	23.4	5.0	23.8
6 months	6.8	29.4	13.5	35.5	6.2	29.8	6.7	30.3
1 year	9.5	38.2	16.9	44.8	8.5	38.2	9.4	39.0
2 years	12.8	49.8	21.1	56.6	12.0	49.6	13.4	50.9
3 years	14.9	58.1	23.6	64.8	14.3	59.1	15.9	60.4
4 years	16.6	64.9	25.4	71.2	15.9	66.3	17.8	67.7
5 years	18.0	70.3	26.8	76.0	17.4	72.1	19.3	73.4
6 years	18.9	74.5	27.5	79.7	18.0	76.2	19.9	77.5

Abbreviations: CABG, coronary artery bypass grafting; IS, ischemic stroke; MI, myocardial infarction; PCI, percutaneous coronary intervention; UA, unstable angina.

Patterns for the cumulative incidence of death after an index event were similar for the 2008 MI and IS cohorts (Figure 1C). After an index MI event, the cumulative incidence of death was 25.0% at 6 months, 32.3% at 1 year, 51.4% at 3 years, and 69.2% at 6 years. After an index IS event, the cumulative incidence of death was 25.5% at 6 months, 32.7% at 1 year, 51.9% at 3 years, and 70.2% at 6 years.

Patients with diabetes accounted for 47.2% (12 526/26 548) of the 2012 MI cohort and 42.5% (7539/17 728) of the IS cohort (Table S1). Patients with diabetes were younger and more likely to be non‐white compared with those without diabetes. Patients with diabetes also had significantly (*P* < 0.0001) higher prevalence of key comorbid conditions, including chronic kidney disease (MI cohort: 59.1% with diabetes, 40.4% without diabetes; IS cohort: 43.7% with diabetes, 28.3% without diabetes) and peripheral artery disease (MI cohort: 33.3% with diabetes, 24.5% without diabetes; IS cohort: 27.3% with diabetes, 22.4% without diabetes; Table S1).

**Figure 2:**
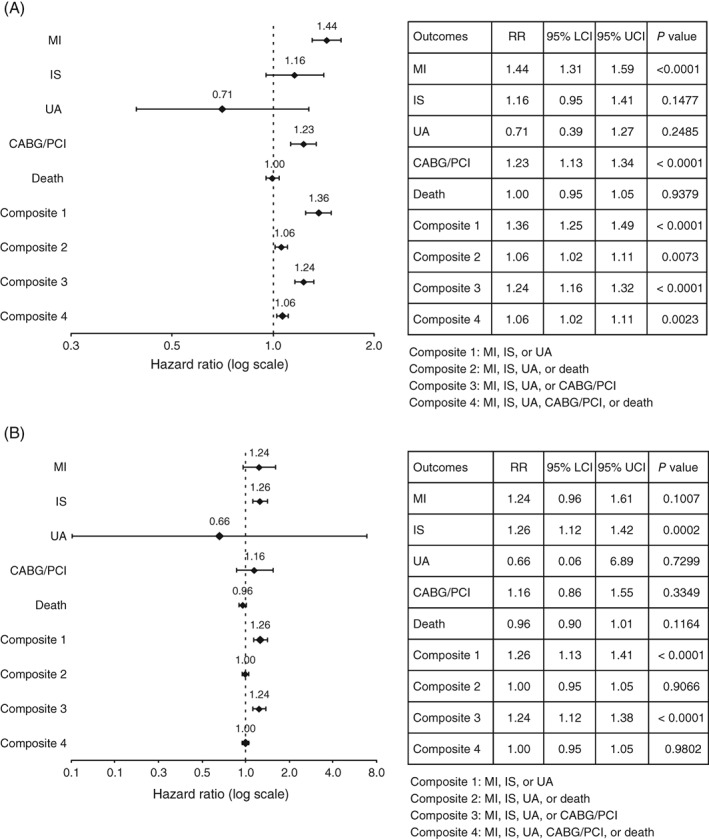
Adjusted risk ratios of outcomes associated with diabetes: 2012 (A) MI and (B) IS cohorts. CABG, coronary artery bypass grafting; IS, ischemic stroke; LCI, lower confidence interval; MI, myocardial infarction; PCI, percutaneous coronary intervention; RR, risk ratio; UA, unstable angina; UCI, upper confidence interval

In the MI cohort, diabetes was significantly associated with higher MI recurrence (adjusted risk ratio [ARR], 1.44; 95% confidence interval [CI], 1.31‐1.59) and risk of CABG/PCI (ARR, 1.23; 95% CI, 1.13‐1.34; Figure 2A). In the IS cohort, diabetes was significantly associated with higher IS recurrence (ARR, 1.26; 95% CI, 1.12‐1.42; Figure 2B). Diabetes was not significantly associated with all‐cause death in either disease cohort. Risks for composite measures that included MI, IS, or UA and MI, IS, UA, or CABG/PCI were significantly higher for patients with diabetes in both the MI and IS cohorts; however, composite measures that included death were significant only in the MI cohort. The number and percentage of patients with at least one CVD event following the index event is presented by diabetes status in Table S2.

## 4 DISCUSSION

The results of the present analysis indicate that the first year following an event of MI or IS represents a critical period for potential secondary prevention in older patients with CVD. We observed a high rate of MI and IS recurrence in the first year of survival after an MI or IS (7.7% and 6.7%, respectively), and the risk of recurrence persisted and continued to increase for up to 6 years of follow‐up (14.3% and 13.4%, respectively). These findings confirm results from other retrospective observational studies, in which the risk of recurrent events was highest in the first year but remained elevated in subsequent years.^17^, ^18^ Moreover, many patients in this Medicare sample experienced multiple recurrent events (11.6 and 10.2 events per 100 patient‐years for MI and IS, respectively, in the 2012 cohort).

Although multiple event rates are not commonly reported in clinical trials, the occurrence of multiple events is an important factor in the overall assessment of cardiovascular burden. One recent publication categorized cardiovascular events following acute coronary syndrome (ACS) in four antithrombotic therapy clinical trials (N = 4307).^19^ In this secondary prevention setting of stabilized patients who did not have a cardiovascular event within the first 7 days after hospitalization for ACS, 9.2% experienced an ischemic event within approximately 1 year post‐ACS, and 32% of those with a non‐fatal first event experienced a second event. History of stroke/TIA was associated with a significantly greater risk for subsequent stroke (hazard ratio, 2.36). For patients having a second event, the second event typically occurred soon after the first post‐ACS MI or stroke. In a recent sub‐analysis of the FOURIER trial, 1999 of the 4906 total events reported during 2.2 years of exposure were multiple recurrent events.^3^ Patients in FOURIER were younger and were receiving moderate‐ or high‐intensity statin therapy; thus, higher multiple recurrence rates might be expected among older patients in real‐world clinical practice.

The prevalence of comorbidities such as hypertension and diabetes, was high in the study population, and as shown in other studies, diabetes is a significant risk factor for recurrent events.^17^, ^18^, ^19^, ^20^, ^21^ In the Hoorn cohort study, the incidence of a recurrent cardiovascular event during approximately 4 years of follow‐up was 60% higher for individuals with diabetes compared with those without, and higher glycated hemoglobin was one of the predictive risk factors for a recurrent event.^21^ A recent systematic review of morbidity and mortality in patients who survived for at least 1 year following MI found that older age, history of stroke, and comorbidities, including diabetes, hypertension, peripheral artery disease, and renal disease, were associated with a greater risk of recurrent cardiovascular events or death.^20^

Overall, these findings highlight the need for clinicians to closely monitor and aggressively treat these high‐risk patients after discharge for MI or IS to ensure that relevant prognostic factors such as lipid, glucose, and blood pressure levels are achieved according to guidelines.^22^, ^23^, ^24^, ^25^, ^26^ For example, the 2018 guidelines for early management of acute IS recommend the measurement of blood cholesterol levels for patients who have an IS of atherosclerotic origin while on optimized statin therapy, as these patients could be candidates for treatment with a proprotein convertase subtilisin/kexin type 9 inhibitor to reduce the risk of subsequent cardiovascular‐related death, MI, or stroke.^26^ However, challenges to managing CVD and its comorbidities in older patients include the risk of hypoglycemia with aggressive treatment of diabetes; risk of orthostatic hypotension and falls with aggressive treatment of hypertension; and myalgia and patient complaints of cognitive decline with statin treatment for hyperlipidemia.^22^, ^23^, ^27^ Although statins are a mainstay of treatment for hyperlipidemia and primary prevention of CVD for many patients, some studies have shown lower‐than‐expected statin treatment rates for older patients with CVD in the primary care setting.^28^, ^29^ In addition, low adherence to high‐intensity statin therapy following MI has been demonstrated in a Medicare sample; approximately 60% of the patients had down‐titrated, low adherence to, or discontinued statin therapy within 2 years.^30^

Approximately, one‐third of patients in our study cohort died within 1 year of the index MI or IS, and approximately half died within 3 years. These high all‐cause death rates may reflect the advanced age, history of CVD, and prevalence of comorbid conditions in the study population and are relatively consistent with other studies in similar populations. For example, 1‐year all‐cause mortality rates (2010) for Medicare patients who experienced a recurrent MI or stroke within 1 year of hospitalization for MI were 30% for patients with recurrent MI, 35% for those with IS following MI, and 61% for those with hemorrhagic stroke following MI.^31^, ^32^ In another study of MI survivors at least 65 years old who subsequently died within 3 years of follow‐up, >50% of the deaths were attributed specifically to CVD.^18^ Although the presence of diabetes was not associated with an increased risk of death in the present study, a 1.5‐fold increase in absolute long‐term mortality risk after MI has been reported for patients with diabetes compared with those without.^33^

This study has some important limitations. First, it was an observational study using a 20% Medicare random sample; therefore, the severity of illness could not be fully controlled due to possible residual confounding. Likewise, lipid levels and cardiovascular‐related death rates were not available in the Medicare dataset; however, the presence of hypercholesterolemia is highly likely in the study population, since all patients had MI or IS of atherosclerotic or ischemic origin. Although recurrence rates could be affected by CVD medication use, we did not attempt to stratify by medication use because the analysis would be biased without also considering lipid values. In addition, the population included secondary prevention patients who had at least one prior event of MI or IS in 2008 or 2012; therefore, the index event was not necessarily the first event of MI or IS. Finally, the findings may not be generalizable to the entire United States population, including individuals who are not Medicare beneficiaries. Areas of future research could include following a real‐world cohort of patients after their first cardiovascular event to characterize potential predictors and protectors of recurrence.

## 5 CONCLUSIONS

This sample of Medicare beneficiaries who survived an event of MI or IS experienced high rates of recurrent cardiovascular events and high mortality rates in the first survival year. These high event rates persisted in subsequent years, patients experienced multiple recurrent events during the first year, and diabetes increased the risk of recurrent cardiovascular events. The clinical implications highlight the need for aggressive intervention in all aspects of secondary prevention of CVD in older high‐risk patients, including the management of common comorbidities. Older patients with diabetes, in particular, may require more intensive and comprehensive monitoring. The recurrent event rates after MI or IS in this study provide real‐world estimates of disease burden in older patients with CVD and potentially could be used for planning future clinical trials that assess CVD burden.

## CONFLICTS OF INTEREST

All authors from the Chronic Disease Research Group, which is a program of the Hennepin Healthcare Research Institute, had full access to all study data and take responsibility for the integrity of data and the accuracy of data analysis. Drs Li, Peng, Guo, and Herzog, and Ms Wang are employees of the Chronic Disease Research Group, which is under contract with and has received funding from Amgen Inc. to conduct this research. Drs Qian, Xiang, and Lopez are employees and stockholders of Amgen Inc. Ms Wade reports consulting fees from Amgen Inc. Dr Herzog reports research grants from Amgen Inc. and ZOLL; consulting fees from AbbVie, FibroGen, Relypsa, OxThera, Sanifit, and ZS Pharma; royalties from UpToDate; stock at Merck; and employment at Hennepin Health System. Dr Handelsman reports research grants and consultant and speaker honoraria from Aegerion, Amarin, Amgen, AstraZeneca, Bristol‐Myers Squibb, Boehringer Ingelheim, Boehringer Ingelheim‐Lilly, Gan & Lee, Gilead, Grifols, Hanmi, Intarcia, Janssen, Lexicon, Lilly, Merck, Mylan, Merck‐Pfizer, Novo Nordisk, Regeneron, and Sanofi.

## Supporting information


**FIGURE S1** Study design schema: MI cohort example. Abbreviations: CABG, coronary artery bypass grafting; ESRD, end‐stage renal disease; IS, ischemic stroke; MI, myocardial infarction; PCI, percutaneous coronary intervention; UA, unstable angina
**FIGURE S2** Selection of patients with MI or IS in the Medicare 20% sample: 2012 cohort. Abbreviations: ESRD, end‐stage renal disease; IS, ischemic stroke; MI, myocardial infarction
**TABLE S1** Characteristics of patients with atherosclerotic cardiovascular disease by baseline diabetes status: 2012 cohort
**TABLE S2** Number and percentage of patients with ≥1 event during follow‐up: 2012 cohort
**APPENDIX SA** Definitions for myocardial infarction, ischemic stroke, and diabetes
**APPENDIX SB** Definitions for outcome events
**APPENDIX SC** Definitions for comorbid conditions
