# Supplementary material for: Cardiovascular events and death after myocardial infarction or ischemic stroke in an older Medicare population
Source: Clin Cardiol. 2019 Feb 25;42(3):391–9. doi: 10.1002/clc.23160 (PMC6712383; doi:10.1002/clc.23160)
Supplement: Supplementary file 1 — FIGURE S1 Study design schema: MI cohort example. Abbreviations: CABG, coronary artery bypass grafting; ESRD, end‐stage renal disease; IS, ischemic stroke; MI, myocardial infarction; PCI, percutaneous coronary intervention; UA, unstable angina FIGURE S2 Selection of patients with MI or IS in the Medicare 20% sample: 2012 cohort. Abbreviations: ESRD, end‐stage renal disease; IS, ischemic stroke; MI, myocardial infarction TABLE S1 Characteristics of patients with atherosclerotic cardiovascular disease by baseline diabetes status: 2012 cohort TABLE S2 Number and percentage of patients with ≥1 event during follow‐up: 2012 cohort APPENDIX SA Definitions for myocardial infarction, ischemic stroke, and diabetes APPENDIX SB Definitions for outcome events APPENDIX SC Definitions for comorbid conditions [file CLC-42-391-s001.docx]

**Supplementary data**


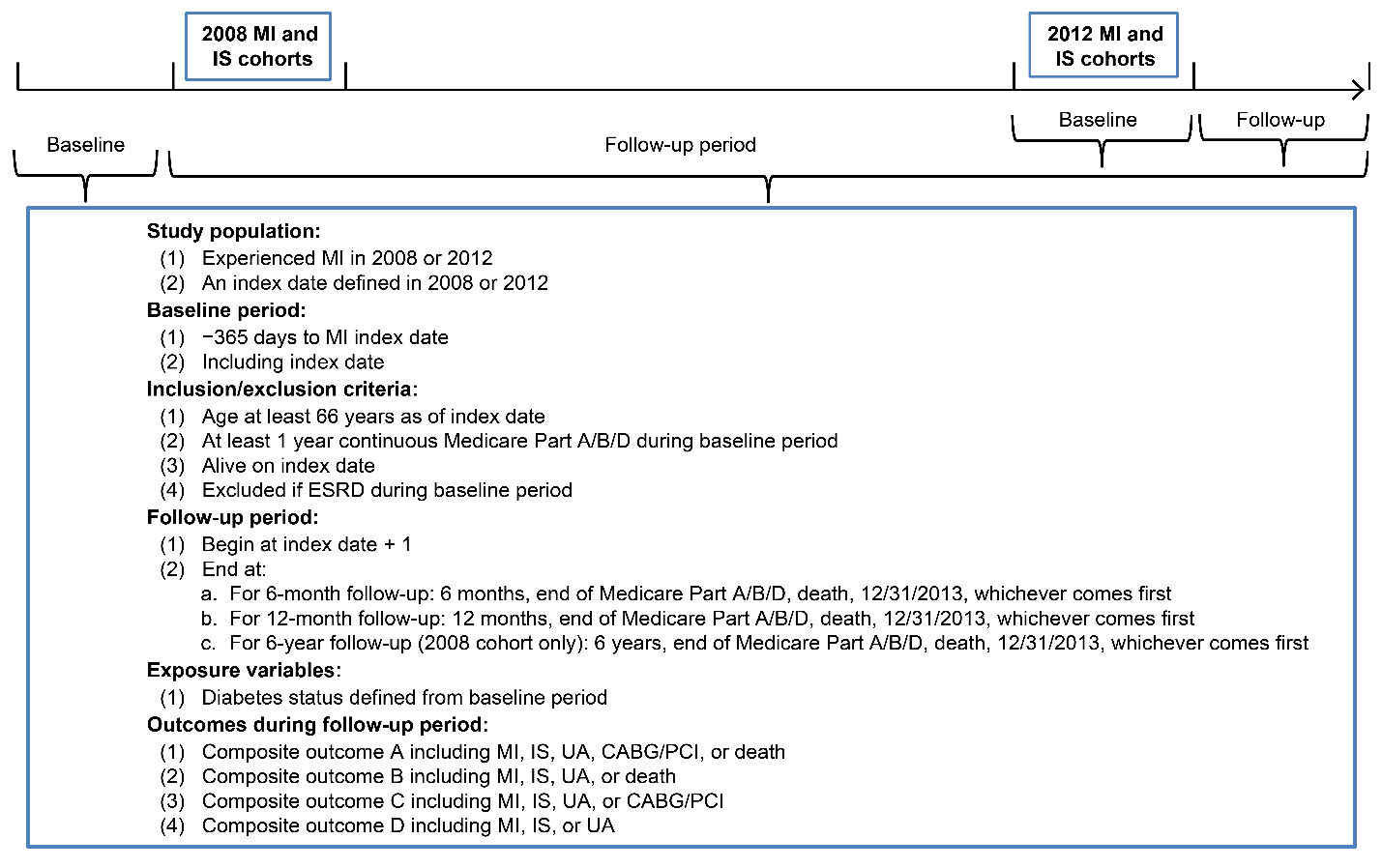


**SUPPLEMENTARY FIGURE S1** Study design schema: MI cohort example. Abbreviations: CABG, coronary artery bypass grafting; ESRD, end-stage renal disease; IS, ischemic stroke; MI, myocardial infarction; PCI, percutaneous coronary intervention; UA, unstable angina


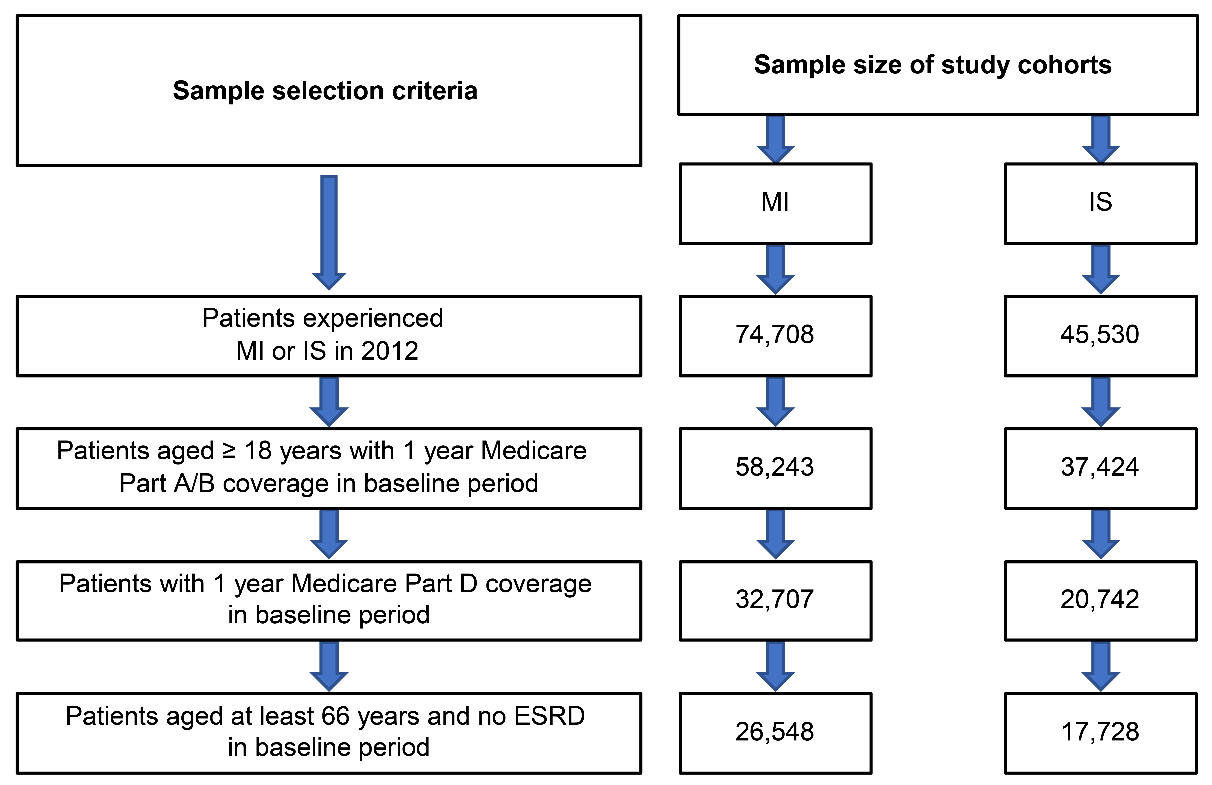


**SUPPLEMENTARY FIGURE S2** Selection of patients with MI or IS in the Medicare 20% sample: 2012 cohort. Abbreviations: ESRD, end-stage renal disease; IS, ischemic stroke; MI, myocardial infarction

**SUPPLEMENTARY TABLE S1** Characteristics of patients with atherosclerotic cardiovascular disease by baseline diabetes status: 2012 cohort

| Study Cohorts | MI | | | IS | | |
| --- | --- | --- | --- | --- | --- | --- |
|  | With Diabetes  (N = 12,526) | Without Diabetes  (N = 14,022) | *P* Value | With Diabetes  (N = 7539) | Without Diabetes  (N = 10,189) | *P* Value |
| Demographics |  |  |  |  |  |  |
| Age, years, mean (SD) | 78.8 (7.8) | 81.2 (8.6) | – | 79.2 (7.8) | 82.2 (8.2) | – |
| Age group on index date, % |  |  |  |  |  |  |
| 66-74 years | 36.7 | 28.6 | < .0001 | 34.4 | 23.2 | < .0001 |
| 75-84 years | 39.2 | 35.2 |  | 40.1 | 36.6 |  |
| 85 years or older | 24.1 | 36.2 |  | 25.5 | 40.2 |  |
| Race, % |  |  |  |  |  |  |
| White | 80.7 | 88.8 | < .0001 | 75.5 | 86.2 | < .0001 |
| Black | 10.9 | 6.6 |  | 16.1 | 8.7 |  |
| Other/unknown | 8.4 | 4.6 |  | 8.4 | 5.1 |  |
| Gender, % |  |  |  |  |  |  |
| Male | 43.2 | 40.9 | < .0001 | 38.2 | 33.7 | < .0001 |
| Female | 56.8 | 59.2 |  | 61.8 | 66.3 |  |
| Census region, % |  |  |  |  |  |  |
| Northeast | 20.9 | 20.4 | .0009 | 17.2 | 17.3 | .0011 |
| Midwest | 24.4 | 26.3 |  | 23.6 | 25.2 |  |
| South | 40.5 | 39.0 |  | 44.7 | 42.3 |  |
| West | 13.9 | 14.2 |  | 14.2 | 15.1 |  |
| Missing | 0.2 | 0.1 |  | 0.3 | 0.2 |  |
| Comorbidity prevalence, % |  |  |  |  |  |  |
| MI (STEMI or NSTEMI) | 38.0 | 29.6 | < .0001 | 18.9 | 13.8 | < .0001 |
| Unstable angina | 7.7 | 4.9 | < .0001 | 2.7 | 1.8 | < .0001 |
| Ischemic stroke | 7.8 | 6.0 | < .0001 | 27.1 | 23.5 | < .0001 |
| Hemorrhagic stroke | 1.3 | 1.2 | .5328 | 5.1 | 4.6 | .128 |
| Cerebrovascular disease | 25.6 | 19.4 | < .0001 | 67.8 | 61.7 | < .0001 |
| TIA | 3.3 | 3.1 | .2245 | 10.0 | 9.3 | .1458 |
| Carotid endarterectomy | 0.8 | 0.6 | .049 | 2.0 | 2.0 | .877 |
| Carotid/vertebral/basilar stenting | 0.2 | 0.1 | .2525 | 0.6 | 0.6 | .8072 |
| CABG surgery, PCI | 35.1 | 34.2 | .0963 | 3.9 | 2.1 | < .0001 |
| PAD | 33.3 | 24.5 | < .0001 | 27.3 | 22.4 | < .0001 |
| PAD with amputation *or* peripheral artery bypass *or* peripheral angioplasty | 3.3 | 1.6 | < .0001 | 1.9 | 1.0 | < .0001 |
| Aneurysm | 3.9 | 5.6 | < .0001 | 2.7 | 3.8 | < .0001 |
| Endovascular stent/graft | 2.0 | 1.8 | .273 | 0.4 | 0.5 | .5896 |
| Hypertension | 96.5 | 88.8 | < .0001 | 97.7 | 92.2 | < .0001 |
| Dyslipidemia/hyperlipidemia | 29.5 | 21.2 | < .0001 | 27.6 | 22.1 | < .0001 |
| Heart failure | 63.7 | 49.5 | < .0001 | 39.1 | 27.7 | < .0001 |
| Venous thromboembolism | 7.5 | 6.7 | .0119 | 6.9 | 6.1 | .0289 |
| Coronary atherosclerosis/angina/old MI | 84.9 | 76.5 | < .0001 | 53.6 | 39.2 | < .0001 |
| HeFH | 0.6 | 0.4 | .0045 | 0.5 | 0.3 | .0188 |
| Cancer (excluding nonmelanoma skin cancer) | 13.5 | 14.5 | .0189 | 12.2 | 12.7 | .3238 |
| HIV | 0.1 | –^a^ | .122 | –^a^ | 0.1 | .8192 |
| Rheumatoid arthritis | 3.6 | 4.2 | .0117 | 3.5 | 3.6 | .6367 |
| CKD stages 1-5 (not on dialysis or transplant) | 59.1 | 40.4 | < .0001 | 43.7 | 28.3 | < .0001 |
| Carotid/vertebral/basilar stenosis | 10.0 | 7.5 | < .0001 | 24.0 | 21.3 | < .0001 |
| Smoking | 33.0 | 33.3 | .6274 | 27.1 | 26.9 | .8377 |
| Obesity^b^ | 15.1 | 4.5 | < .0001 | 12.5 | 3.8 | < .0001 |

Abbreviations: CABG, coronary artery bypass grafting; CKD, chronic kidney disease; HeFH, heterozygous familial hypercholesterolemia; HIV, human immunodeficiency virus; IS, ischemic stroke; MI, myocardial infarction; NSTEMI, non–ST-elevation MI; PAD, peripheral arterial disease; PCI, percutaneous coronary intervention; SD, standard deviation; STEMI, ST-elevation MI; TIA, transient ischemic attack.

^a^ Value is suppressed due to smaller event size (10 events or less) according to Centers for Medicare & Medicaid Services reporting rules.

^b^ Obesity was defined by International Classification of Diseases, Ninth Revision, Clinical Modification diagnosis codes: 278.01, 278.03, V85.3x, and V85.4x.

**SUPPLEMENTARY TABLE S2** Number and percentage of patients with ≥ 1 event during follow-up: 2012 cohort

|  | MI | IS | UA | CABG/PCI | Death |
| --- | --- | --- | --- | --- | --- |
| All patients with MI, n (%) | 2514 (7.7) | 541 (1.7) | 81 (0.3) | 3281 (10.0) | 9977 (30.5) |
| With diabetes | 1611 (9.7) | 312 (1.9) | 48 (0.3) | 1961 (11.8) | 5316 (32.1) |
| Without diabetes | 903 (5.6) | 229 (1.4) | 33 (0.2) | 1320 (8.4) | 4661 (28.9) |
| All patients with IS, n (%) | 328 (1.6) | 1427 (6.9) | –^a^ | 311 (1.5) | 6247 (30.1) |
| With diabetes | 195 (2.1) | 773 (8.2) | –^a^ | 193 (2.0) | 2815 (29.8) |
| Without diabetes | 133 (1.2) | 654 (5.8) | –^a^ | 118 (1.1) | 3432 (30.4) |

Abbreviations: CABG, coronary artery bypass grafting; IS, ischemic stroke; MI, myocardial infarction; PCI, percutaneous coronary intervention; UA, unstable angina.

^a^ Value is suppressed due to smaller event size (10 events or less) according to Centers for Medicare & Medicaid Services reporting rules.

## SUPPLEMENTARY APPENDIX A Definitions for myocardial infarction, ischemic stroke, and diabetes

| Disease | ICD-9-CM Diagnosis Codes Used | Disease Definition Algorithm (During Cohort Year) |
| --- | --- | --- |
| Myocardial infarction | 410.xx (excluding 410.x2 for index event) | 1 inpatient claim, any position (discharge date as index date) |
| Ischemic stroke | 433.x1, 434.x1 | 1 inpatient claim, any position (discharge date as index date) |
| Diabetes | 250.x0, 250.x2 | 1 inpatient or 2 outpatient claims, any position, at least 30 days apart within 1 year prior to index date |

Abbreviations: ICD-9-CM, International Classification of Diseases, Ninth Revision, Clinical Modification.

**SUPPLEMENTARY APPENDIX B** Definitions for outcome events

| Outcome | Definition |
| --- | --- |
| Myocardial infarction | 410.xx; primary position |
| Unstable angina | 411.1, 411.81, 411.89; primary position |
| Ischemic stroke | 433.x1, 434.x1; primary position |
| Transient ischemic attack | 435.xx; primary position |
| Coronary artery bypass grafting/percutaneous coronary intervention | ICD-9-CM procedure codes: 3610, 3611, 3612, 3613, 3614, 3615, 3616, 3617, 3619, 36.0X, 0066, 362, 363; any claim source and any position  -OR- CPT codes: 33510-33523, 33533-33536, 92982, 92995, G0290, G0291, 92980, 92981; any claim source and any position |

Abbreviations: CPT, Current Procedural Terminology; ICD-9-CM, International Classification of Diseases, Ninth Revision, Clinical Modification.

**SUPPLEMENTARY APPENDIX C** Definitions for comorbid conditions

| Condition/Event | Codes Used | Algorithm |
| --- | --- | --- |
| MI (STEMI or NSTEMI) | 410.xx, 412 | 1 IP or 2 OP claims at least 30 days apart within 1 year prior to index date; any position |
| Unstable angina | 411.1, 411.81, 411.89 | 1 IP or 2 OP claims at least 30 days apart within 1 year prior to index date; any position |
| Ischemic stroke | 433.x1, 434.x1 | 1 IP or 2 OP claims at least 30 days apart within 1 year prior to index date; any position |
| Hemorrhagic stroke | 430, 431, 432 | 1 IP or 2 OP claims at least 30 days apart within 1 year prior to index date; any position |
| Cerebrovascular disease | 436, 437, 438, 433.x0, 434.x0 | 1 IP or 2 OP claims at least 30 days apart within 1 year prior to index date; any position |
| TIA | 435.x | 1 IP or 2 OP claims at least 30 days apart within 1 year prior to index date; any position |
| Carotid endarterectomy | ICD-9-CM procedure code: 38.12 -OR- CPT code: 35301 | 1 IP procedure -OR- 1 CPT within 1 year prior to index date; any position |
| Carotid/vertebral/basilar stenting | ICD-9-CM procedure codes: 00.61, 00.62, 00.63, 00.64, 00.65 -OR- CPT codes: 37215, 37216, 37218 | 1 IP procedure -OR- 1 CPT within 1 year prior to index date; any position |
| CABG surgery, PCI | ICD-CM-9 procedure codes: 3610, 3611, 3612, 3613, 3614, 3615, 3616, 3617, 3619, 36.0X, 0066 -OR- CPT codes: 33510-33523, 33533-33536, 92982, 92995, G0290, G0291, 92980, 92981 | 1 procedure -OR- 1 CPT within 1 year prior to index date; any position; any source |
| PAD | 440.xx, 443.9 | 1 IP or 2 OP claims at least 30 days apart within 1 year prior to index date; any position |
| PAD with amputation  OR peripheral artery bypass  OR peripheral angioplasty | 440.xx, 443.9 AND at least one of the following: | 1 IP or 1 OP; any position |
|  | ICD-9-CM procedure codes: 84.0, 84.1, 84.91, 39.25, 39.26, and 39.29, 00.55, 39.50, 39.90 -OR-  CPT codes: 24900, 24920, 25900, 25905, 25920, 25927, 27295, 27590, 27591, 27592, 27598, 27880, 27881, 27882, 27888, 27889, 28800, 28805, 34900, 35131, 35132, 35141, 35142, 35151, 35152, 34051, 34151, 34201, 34203, 34800-34834, 35081-35103, 35331, 35341, 35351, 35355, 35361, 35363, 35371, 35372, 35381, 35450, 35452, 35454, 35456, 35459, 35470, 35471, 35472, 35473, 35474, 35480, 35481, 35482, 35483, 35485, 35490, 35491, 35492, 35493, 35495, 35521, 35531, 35533, 35541, 35546, 35548, 35549, 35551, 35556, 35558, 35563, 35565, 35566, 35571, 35583, 35585, 35587, 35621, 35623, 35646, 35647, 35651, 35654, 35656, 35661, 35663, 35665, 35666, 35671 | 1 ICD-9-CM procedure -OR- 1 CPT; any position  Requires both ≥ 1 ICD-9-CM and ≥ 1 procedure at any point within lookback |
| Aneurysm | 441.x (AAA), 442.x (other aneurysm) | 1 IP/SNF/HH/HS; any position -OR- 2 OP/PB/DME 30 days apart; any position |
| Endovascular stent/graft | DRG codes: 237, 238  -OR-  CPT codes: 34802, 34825, 34826 | 1 IP/SNF DRG -OR- 1 CPT; any position |
| Hypertension | 401.x, 403.0x, 403.1x, 403.9x | 1 IP or 2 OP claims at least 30 days apart within 1 year prior to index date; any position. Medication use not required |
| Dyslipidemia/hyperlipidemia | 272.0 | 1 IP or 2 OP claims at least 30 days apart within 1 year prior to index date; any position |
| Heart failure | 398.91, 402.01, 402.11, 402.91, 404.03, 404.11, 404.13, 404.91, 404.93, 425.4x-425.9x, 428.x | 1 IP or 2 OP claims at least 30 days apart within 1 year prior to index date; any position |
| Venous thromboembolism | 415.xx, 451.xx, 453.xx | 1 IP or 2 OP claims at least 30 days apart within 1 year prior to index date; any position |
| Coronary atherosclerosis/angina/old MI | 414.0x (coronary atherosclerosis), 413.x (stable angina), 410.x2 (second visit event after the first one), 412 (old MI) | 1 IP/SNF/HH/HS; any position (index date = discharge date) -OR- 2 OP/PB/DME at least 30 days apart; any position (index date = second fromdate) |
| HeFH | 272.0; medications | 1 IP/SNF/HH/HS/OP/PB/DME ICD-9-CM diagnosis; any position (index date = discharge date for IP/SNF/HH/HS = fromdate for OP/PB/DME) -AND- (high-intensity statin use and concurrent prescription for ezetimibe -OR- high-intensity statin use and concurrent use of ≥ 2 other medications to lower LDL-C other than ezetimibe) |
| Cancer (excluding nonmelanoma skin cancer) | 140.xx-172.xx, 174.xx-195.xx, 200x-208.x, 196.xx-198.xx, 199.1 | 1 IP or 2 OP separated by at least 30 days; any position |
| HIV | 042, V08; medications | For 042: 1 IP/SNF/HH/HS; any position -OR- 2 OP/PB/DME; any position -OR- For V08: any source; any position  -AND- ≥ 2 medications (index date = fulfill all criteria) |
| Rheumatoid arthritis | 714.0 | 1 IP or 2 OP claims at least 30 days apart within 1 year prior to index date; any position |
| CKD (stages 1-5), not on dialysis or transplant | 016.0x, 095.4, 223.0, 250.4x, 274.1x, 271.4, 283.11, 285.21, 403.x1, 404.02, 404.03, 404.12, 404.13, 404.92, 404.93, 440.1, 442.1, 447.3, 572.4, 580.xx, 581.xx, 582.xx, 583.xx, 587, 584.x, 585.x, 586, 588.xx, 591, 794.4, 646.2x, 642.1x, 753.12, 753.13, 753.14, 753.15, 753.16, 753.17, 753.19, 753.20 | 1 IP or 2 OP claims at least 30 days apart within 1 year prior to index date; any position |
| Carotid/vertebral/basilar stenosis | 433.1x (carotid), 433.2x (vertebral), 433.0x (basilar) | 1 IP or 2 OP separated by at least 30 days; any position |
| Smoking | 305.1; V15.82 | 1 IP or 2 OP separated by at least 30 days; any position |
| Obesity | 278.01; 278.03; v85.3x; v85.4x | 1 IP or 2 OP separated by at least 30 days; any position |

Abbreviations: AAA, abdominal aortic aneurysm; CABG, coronary artery bypass grafting; CKD, chronic kidney disease; CPT, Current Procedural Terminology; DME, durable medical equipment; DRG, diagnosis-related group; HeFH, heterozygous familial hypercholesterolemia; HH, home health; HIV, human immunodeficiency virus; HS, hospice; ICD-9-CM, International Classification of Diseases, Ninth Revision, Clinical Modification; IP, inpatient; LDL-C, low‑density lipoprotein cholesterol; MI, myocardial infarction; NSTEMI, non–ST-elevation MI; OP, outpatient; PAD, peripheral artery disease; PB, Part B; PCI, percutaneous coronary intervention; SNF, skilled nursing facility; STEMI, ST-elevation MI; TIA, transient ischemic attack.
